# Altered Glycolysis and Mitochondrial Respiration in a Zebrafish Model of Dravet Syndrome^1^,^2^,^3^

**DOI:** 10.1523/ENEURO.0008-16.2016

**Published:** 2016-04-05

**Authors:** Maneesh G. Kumar, Shane Rowley, Ruth Fulton, Matthew T. Dinday, Scott C. Baraban, Manisha Patel

**Affiliations:** 1 Department of Pharmaceutical Sciences, University of Colorado Anschutz Medical Campus, Aurora, Colorado 80045; 2 Department of Neurological Surgery, University of California San Francisco, San Francisco, California 94143

**Keywords:** Dravet syndrome, epilepsy, glycolysis, metabolism, mitochondrial respiration, zebrafish

## Abstract

Altered metabolism is an important feature of many epileptic syndromes but has not been reported in Dravet syndrome (DS), a catastrophic childhood epilepsy associated with mutations in a voltage-activated sodium channel, Nav1.1 (SCN1A). To address this, we developed novel methodology to assess real-time changes in bioenergetics in zebrafish larvae between 4 and 6 d postfertilization (dpf). Baseline and 4-aminopyridine (4-AP) stimulated glycolytic flux and mitochondrial respiration were simultaneously assessed using a Seahorse Biosciences extracellular flux analyzer. *Scn1Lab* mutant zebrafish showed a decrease in baseline glycolytic rate and oxygen consumption rate (OCR) compared to controls. A ketogenic diet formulation rescued mutant zebrafish metabolism to control levels. Increasing neuronal excitability with 4-AP resulted in an immediate increase in glycolytic rates in wild-type zebrafish, whereas mitochondrial OCR increased slightly and quickly recovered to baseline values. In contrast, *scn1Lab* mutant zebrafish showed a significantly slower and exaggerated increase of both glycolytic rates and OCR after 4-AP. The underlying mechanism of decreased baseline OCR in *scn1Lab* mutants was not because of altered mitochondrial DNA content or dysfunction of enzymes in the electron transport chain or tricarboxylic acid cycle. Examination of glucose metabolism using a PCR array identified five glycolytic genes that were downregulated in *scn1Lab* mutant zebrafish. Our findings in *scn1Lab* mutant zebrafish suggest that glucose and mitochondrial hypometabolism contribute to the pathophysiology of DS.

## Significance Statement

These studies demonstrate that metabolism can be studied in zebrafish, and that this novel approach can be used: (1) to evaluate chemoconvulsant or genetic zebrafish models of epilepsy, or (2) in metabolism-based drug screening efforts to identify compounds that modulate glycolysis or mitochondrial function. As more models of epilepsy become available, the array of techniques demonstrated here can be used to rapidly characterize metabolic contributions to disease states.

## Introduction

Glycolysis and mitochondrial oxidative phosphorylation are key energy producing pathways in the brain. These metabolic pathways may play a role in the control of seizures and epileptogenesis (Rowley et al., 2015). For example, glycolytic rates are acutely increased during ictal activity (Stafstrom et al., 2008) and ictal hypermetabolism in human epileptic foci is followed by interictal hypometabolism, probably resulting from a decreased mitochondrial bioenergetic capacity (Chugani et al., 1994; Lee et al., 2012). Hypometabolism associated with seizures may reflect glucose transport abnormalities or defects in electron transport chain (ETC) enzymes (Tenney et al., 2014). Seizures are also associated with an imbalance in ATP levels (Grisar, 1984) and represent a common symptom in patients with mitochondrial disease (Wallace et al., 1988; Mecocci et al., 1993; Liang et al., 2012).

Although altered metabolism may be a feature of acquired and/or focal epilepsies, it has not been systematically investigated in any genetic form of epilepsy. Dravet syndrome (DS) is one example of a severe genetic epilepsy most commonly associated with *de novo* mutations in a brain-specific voltage-activated sodium channel (SCN1A). DS children exhibit significant developmental delays, cognitive deficits, behavioral disturbances, and increased risk of sudden unexpected death in epilepsy (SUDEP; Dravet, 2011). Two types of observations suggest that metabolic dysfunction may be occurring in DS. First, mitochondrial defects in muscle biopsies have been reported in patients. Second, some DS children respond positively to treatment with ketogenic diets (KDs; Caraballo, 2011). Although numerous mechanisms may underlie the efficacy of KDs (Gano et al., 2014), these observations suggest that energy metabolism is not well studied in DS, or any form of genetic epilepsy. Here we developed novel techniques to show, for the first time, that glycolysis and mitochondrial respiration are abnormal in a zebrafish model of DS and its rescue by a form of a KD. Moreover, altered metabolism in *scn1Lab* mutants was accompanied by downregulation of several glycolytic genes rather than defects in select mitochondrial enzyme activities.

## Materials and Methods

### Animal care

*Scn1Lab* mutant zebrafish were obtained from the Baraban laboratory at the University of California San Francisco (UCSF) and bred in the University of Colorado Anschutz Medical Campus (UCD) zebrafish core facility. When obtained from UCSF, eggs were shipped overnight and immediately placed in a 28.5°C incubator upon arrival. Zebrafish larvae were maintained in “embryo medium” consisting of 0.03% Instant Ocean (Aquarium Systems) in deionized water containing 0.2 ppm methylene blue as a fungicide with no additional glucose anapleurotic substrates added. Homozygous mutants (sorted based on pigmentation) and age-matched sibling larvae were used at 4–6 d postfertilization (dpf). Pentylenetetrazole (PTZ; Sigma-Aldrich) was dissolved in embryo medium, pH balanced to 7.4, and bath applied. KD water was prepared by sonication of 200 µm palmitate (Sigma-Aldrich) and laurate (Sigma-Aldrich) in embryo media containing 100 µm phosphatidyl choline (Sigma-Aldrich) and bath applied (Taylor et al., 2004).

### Metabolic measurements

Glycolysis and mitochondrial respiration rates were simultaneously measured in live zebrafish in an XF24 or XF24e analyzer (Seahorse Bioscience). One fish was loaded per well of a 24-well islet plate and mesh screen placed to hold the zebrafish in place. 4-AP (Sigma-Aldrich) was prepared in embryo medium at a stock concentration of 40 mm and pH balanced to ∼7.4. 4-AP was injected by the XF24 analyzer at a final concentration of 4 mm.

### Behavioral seizure analysis

Zebrafish were placed individually in 96-well Falcon culture dishes. Each well contained ∼75 μl embryo media and one 5 dpf WT zebrafish larvae. Swim behavior was monitored in a DanioVision system, as described previously in the literature (Baraban et al., 2013). Recording sessions (2 min) were analyzed off-line and scored for seizure stage (Baraban et al., 2005) by an investigator blind to the status of the fish.

### Mitochondrial copy number

Relative mitochondrial copy number was determined by real-time PCR (Hunter et al., 2010; Artuso et al., 2012). DNA was extracted from individual fish using the DNeasy Blood and Tissue Kit (Qiagen) following the manufacturer-supplied instructions. A short section of mitochondrial DNA was amplified using Power SYBR green (Forward: 5′-CAAACACAAGCCTCGCCTGTTTAC-3′; Reverse: 5′-CACTGACTTGATGGGGGAGACAGT-3′). This was normalized to the nuclear gene *polg1* (Forward: 5′-GAGAGCGTCTATAAGGAGTAC-3′; Reverse: 5′- GAGCTCATCAGAAACAGGACT-3′). Primers were ordered from Integrated DNA Technologies (Coralville). Fish were exposed to 4-AP for 1 h.

### Enzyme assays

Activity of complexes I–IV was determined from total protein isolates from 25 to 30 pooled zebrafish embryos at 4–6 dpf. Total protein was isolated by resuspending zebrafish in PBS with 0.01% Triton-X and protease inhibitors followed by probe sonication at 30% intensity for three pulses of 5 s each. Lysate was centrifuged at 10,000 rpm for 5 min to remove insoluble material. Total protein concentration was determined by Bradford assay. Complex activity was determined by UV spectrophotometry (Ma et al., 2011). Complex I was determined by the oxidation of NADH at 340 nm. The assay solution consisted of 25 mm potassium phosphate, 5 mm MgCl_2_, 2 mm KCN, 2.5 mg/ml BSA, 0.13 mm NADH, 2 ug/ml antimycin A, and 65 µm ubiquinone1 equilibrated to 30°C. Forty micrograms of total protein was added and activity measured for 5 min. Nonspecific activity was determined by addition of 2 µg/ml of rotenone prior to protein addition. Complex II was determined by reduction of dichlorophenol indophenols at 600 nm. The assay solution consisted of 25 mm KPO_4_, 5 mm MgCl_2_, 2 mm KCN, 20 mm sodium succinate, 50 µm dichlorophenol indophenol, 2 µg/ml rotenone, and 2 µg/ml antimycin A. After equilibrating to 30°C, 40 µg of total protein was added along with 65 µm ubiquinone1 to start the reaction. Activity was determined between 3 and 5 min. Complex III was determined by the reduction of cytochrome C at 550 nm. The assay solution consisted of 50 mm KPO_4_, 1 mm *N*-dodecyl-β-d-maltoside, 2 mm KCN, and 20 mm NADH at 30°C. Forty micrograms of total protein was added along with 100 µm of reduced decylbenzylquinone and 40 µm oxidized cytochrome C to start the reaction. Activity was measured for 3 min. Complex IV was determined by oxidation of cytochrome C at 550 nm. The assay solution consisted of 20 mm KPO_4_ and 0.45 mm *N*-dodecyl-β-d-maltoside equilibrated to 30°C. Forty micrograms of total protein and reduced cytochrome C was added to a final concentration of 15 µm and activity measured for 3 mins.

Activity of select enzymes of the tricarboxylic acid (TCA) cycle, aconitase, fumarase, and malate dehydrogenase, was determined from total protein isolates from 30 pooled zebrafish embryos at 5 dpf. Total protein was isolated as above with the exception of probe sonication at 30% intensity for two pulses of 1 s each. Aconitase and fumarase activity was determined by UV spectrophotometry by following the dehydration of dl-isocitrate or l-malate, respectively, at 240 nm (Patel et al., 1996). The aconitase assay buffer consisted of 50 mm Tris HCl and 600 µm MnCl_2_ at pH 7.4. dl-Isocitrate substrate was added to a final concentration of 20 mm along with 20 µl of total protein. Activity was measured for 3 min. Aconitase activity was inhibited by addition of 0.1 mm potassium ferricyanide. Fumarase assay buffer consisted of 30 mm KH_2_PO_4_ and 100 µm EDTA at a pH of 7.4. l-Malate was added to a final concentration of 5 mm along with 20 µl of total protein. Activity was measured for 3 min. Fumarase activity showed stereospecificity for l-malate; d-malate was inactive. Malate dehydrogenase was determined by measuring the decrease in absorbance at 340 nm resulting from oxidation of NADH. The assay buffer consisted of 0.1 m KPO_4_, 200 µm oxaloacetic acid, and 250 µm NADH, pH 7.4. Five microliters of total protein was added and activity measured for 5 min. All TCA cycle enzyme assays were performed at room temperature.

### Gene expression

Six to eight zebrafish were pooled for each group. RNA was extracted by TRIzol 1 h after 4-AP treatment. cDNA was synthesized using the RT^2^ First Strand Kit (SABiosciences) and hybridized to the Glucose Metabolism PCR array (SABiosciences). Array was analyzed using Applied Biosystem 7500 Real-Time PCR System. Data was analyzed using SABiosciences’ on-line data analysis suite (http://www.sabiosciences.com/pcrarraydataanalysis.php). Array results were verified using specific Taqman primers (Applied Biosystems). Fish were exposed to 4-AP for 1 h.

### Statistics

Statistical methods were performed with GraphPad Prism v6.04 for Windows. Analyses include Holm–Sidak *t* test (for baseline comparisons) and two-way one-way ANOVA with Tukey’s multiple-comparisons test. Behavioral seizure data were analyzed by Shapiro–Wilk normality test followed by Kruskal–Wallis ANOVA on Ranks with Tukey’s multiple comparison *post hoc* test.

## Results

**Figure 1.**
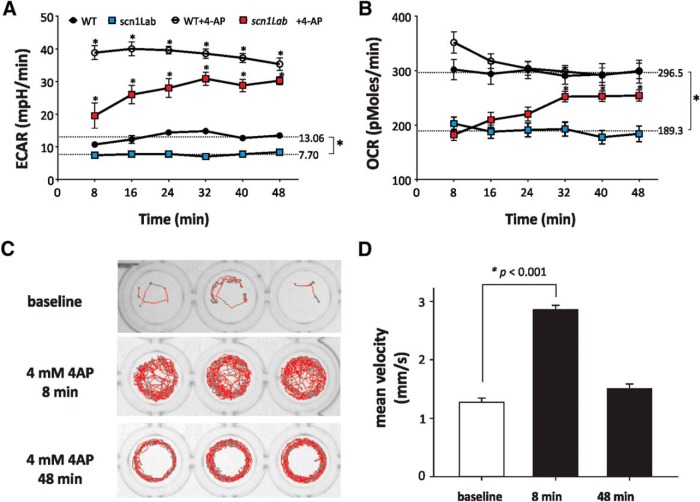
Glycolytic and mitochondrial respiration rates in WT and *scn1Lab* mutant zebrafish at baseline and after 4-AP stimulation. ***A***, ***B***, *Scn1Lab* mutant zebrafish have lower baseline glycolytic and mitochondrial respiration rates than WT zebrafish. 4-AP immediately increases glycolytic and mitochondrial respiration rates in WT zebrafish. *Scn1Lab* mutant zebrafish have a delayed response to 4-AP of ∼30 min. Statistical analysis shows changes relative to time-matched untreated controls; points represent means±S.E.M. *N* = 14 (WT), 16 (*scn1Lab*), 16 (WT+4-AP), 14 (*scn1Lab*+4-AP) individual animals, mean±S.E.M. ***A***, WT vs *scn1Lab*: *p* < 0.0001^a^; WT vs WT+4-AP, *p* = 2.38e-30 (8 min)^o^, *p* = 8.41e-30 (16 min)^p^, *p* = 3.50e-26 (24 min)^q^, *p* = 3.99e-24 (32 min)^r^, *p* = 2.92e-25 (40 min)^s^, *p* = 1.70e-21 (48 min)^t^; *scn1Lab* vs *scn1Lab*+4-AP: *p* = 3.28e-6 (8 min)^u^, *p* = 1.65e-11 (16 min)^v^, *p* = 1.86e-13 (24 min)^w^, *p* = 3.08e-17 (32 min)^x^, *p* = 2.34e-14 (40 min)^y^, *p* = 3.52e-15 (48 min)^z^. ***B***, WT vs *scn1La*: *p* < 0.0001^aa^; *scn1Lab* versus *scn1Lab*+4-AP: *p* = 0.00071 (32 min)^bb^, *p* = 2.20e-5 (40 min)^cc^, *p* = 7.24e-5 (48 min)^dd^. ***C***, ***D***, Locomotion plots for behavioral seizure activity in WT zebrafish exposed to 4 mmm 4-AP. Bar plot showing the mean ± SEM for WT fish at baseline, 8 min after exposure to 4-AP and 48 min after exposure to 4-AP. *N* = 48 WT fish; Kruskal–Wallis one-way ANOVA on ranks with a *post hoc* Tukey test. *p* < 0.05^c,d^ (8 min vs baseline).

To measure real-time glycolytic and mitochondrial respiration rates live zebrafish (4–6 dpf) were placed in an extracellular flux analyzer (Seahorse Biosciences). The analyzer measures extracellular acidification rate (ECAR) and oxygen consumption rate (OCR) in a transient microchamber over 3 min, representing glycolysis and mitochondrial respiration, respectively (Wu, 2009). Baseline glycolysis rates in *scn1Lab* mutant and age-matched WT zebrafish were 7.70 ± 0.18 mpH/min and 13.06 ± 0.61 mpH/min (Fig. 1*A*; Table 1), respectively, indicating a 41% decrease (*p* < 0.0001)^a^ of baseline glycolysis. Similarly, basal OCR was significantly decreased in *scn1Lab* mutant zebrafish (189.3 ± 3.47 pmol/min) compared with WT controls (296.5 ± 1.929 pmol/min, *p* < 0.0001^b^; Fig. 1*B*). Zebrafish obtained from UCSF or bred at UCD and metabolism measured using the Seahorse XF24 or an updated version, the XF24e, showed similar alterations in metabolism between *scn1Lab* and WT siblings (see Fig. 6).

**Table 1.** Statistical table

	Data structure	Type of test	Confidence interval	
a	Normal distribution	*t* test	−6.78 to −3.94	
b	Normal distribution	*t* test	−116.0 to −98.31	
c	Non-normal distribution (Shapiro–Wilk test *p* < 0.05)	One-way ANOVA Kruskal–Wallis on ranks with a *post hoc* Tukey test	*p* < 0.01 ANOVA 8 min vs baseline: *p *< 0.05	
d	Non-normal distribution (Shapiro–Wilk test *p* < 0.05)	One-way ANOVA	Baseline vs 48 min: *p* > 0.05	
e	Normal distribution	Unpaired *t* test	−23.76 to −15.36	
f	Normal distribution	*t* test with Holm–Sidak for multiple comparisons	−245.2 to −110.5	
g	Normal distribution	*t* test with Holm–Sidak for multiple comparisons	−64.78 to −20.95	
h	Normal distribution	One-way ANOVA	WT vs WT+4-AP	−0.9128 to 0.1422
			WT vs *scn1Lab*	−0.5763 to 0.4787
			WT vs *scn1Lab*+4-AP	−0.5664 to 0.4886
i	Normal distribution	Two-way ANOVA	Complex I	−148.8 to 112.6
			Complex II	−138.4 to 123.1
			Complex III	−177.9 to 83.56
			Complex IV	−146.6 to 114.9
j	Normal distribution	Two-way ANOVA	Aconitase	−15.04 to 45.86
			Fumarase	−32.52 to 28.38
			Mal. Dehyd.	−21.06 to 33.41
k	Normal distribution	*t* test	−0.3722 to 3.172	
l	Normal distribution	*t* test	65.33 to 64.27	
m	Normal distribution	*t* test	−236.8 to 132.0	
n	Normal distribution	*t* test	−1.676 to 0.3118	
o	Normal distribution	*t* test with Holm–Sidak for multiple comparisons	−33.97 to −22.24	
p	Normal distribution	*t* test with Holm–Sidak for multiple comparisons	−33.59 to −21.85	
q	Normal distribution	*t* test with Holm–Sidak for multiple comparisons	−31.04 to −19.30	
r	Normal distribution	*t* test with Holm–Sidak for multiple comparisons	−29.59 to −17.86	
s	Normal distribution	*t* test with Holm–Sidak for multiple comparisons	−30.39 to −18.66	
t	Normal distribution	*t* test with Holm–Sidak for multiple comparisons	−27.73 to −16.00	
u	Normal distribution	*t* test with Holm–Sidak for multiple comparisons	−18.01 to −6.278	
v	Normal distribution	*t* test with Holm–Sidak for multiple comparisons	−24.10 to −12.37	
w	Normal distribution	*t* test with Holm–Sidak for multiple comparisons	−26.07 to −14.33	
x	Normal distribution	*t* test with Holm–Sidak for multiple comparisons	−29.68 to −17.95	
y	Normal distribution	*t* test with Holm–Sidak for multiple comparisons	−26.95 to −15.21	
z	Normal distribution	*t* test with Holm–Sidak for multiple comparisons	−27.74 to −16.01	
aa	Normal distribution	*t* test	−116.0 to −98.31	
bb	Normal distribution	*t* test with Holm–Sidak for multiple comparisons	−112.2 to −6.900	
cc	Normal distribution	*t* test with Holm–Sidak for multiple comparisons	−128.0 to −22.77	
dd	Normal distribution	*t* test with Holm–Sidak for multiple comparisons	−122.9 to −17.64	
ee	Normal distribution	One-way ANOVA	Control vs 10 μm	−3.649 to 7.982
			Control vs 50 μm	−6.585 to 5.046
			Control vs 100 μm	−4.812 to 6.819
			Control vs 1 mm	−6.324 to 5.307
			Control vs 4 mM	−7.943 to 2.825

ff	Normal distribution	One-way ANOVA	Control vs 10 μm	−67.77 to 93.79
			Control vs 50 μm	−62.06 to 99.49
			Control vs 100 μm	−39.08 to 122.5
			Control vs 1 mm	−19.22 to 142.3
			Control vs 4 mm	−19.85 to 129.7
gg	Normal distribution	One-way ANOVA	WT vs WT+4-AP	−0.9128 to 0.1422
			WT vs *scn1Lab*	−0.5763 to 0.4787
			WT vs *scn1Lab*+4-AP	−0.5664 to 0.4886
hh	Normal distribution	Two-way ANOVA	Complex I	−148.8 to 112.6
			Complex II	−138.4 to 123.1
			Complex III	−177.9 to 83.56
			Complex IV	−146.6 to 114.9
ii	Normal distribution	Two-way ANOVA	Aconitase	−15.04 to 45.86
			Fumarase	−32.52 to 28.38
			Mal. Dehyd.	−21.06 to 33.41
jj	Normal distribution	*t* test	1.783 to 8.531	
kk	Normal distribution	*t* test	−4.128 to −2.727	
ll	Normal distribution	*t* test	−36.51 to −22.87	
mm	Normal distribution	*t* test	−98.59 to −32.08	
nn	Normal distribution	*t* test	−104.4 to −49.14	
oo	Normal distribution	*t* test	−46.07 to −30.43	
pp	Normal distribution	*t* test	−52.57 to −32.94	
qq	Normal distribution	*t* test	−35.93 to −11.69	
rr	Normal distribution	*t* test	−32.95 to −15.50	

*Scn1Lab* mutant zebrafish have been reported to have seizures very early in life, starting at about 3 dpf (Baraban et al., 2013). To determine the consequences of increased neuronal excitability associated with induced seizure activity, WT zebrafish were stimulated with 4-AP and glycolytic and mitochondrial respiration rates were measured (Figs. 1*A*,*B*). 4-AP, a chemoconvulsant previously shown to induce electrographic seizures in zebrafish (Baraban et al., 2007), increases metabolic demand in cortical cells and synaptosomes (Tibbs et al., 1989; Flynn et al., 2011). Following metabolic challenge with a single, high dose of 4-AP, WT zebrafish showed increased behavioral seizure activity (Fig. 1*C*,*D*) and 360% increase in glycolysis within 8 min. *Scn1Lab* mutants, which exhibit spontaneous seizures at a rate of ∼1 ictal-like event per minute (Baraban et al., 2013), also showed increased glycolytic rate after 4-AP stimulation albeit in a delayed manner, achieving maximal glycolytic rates about 30 min after 4-AP addition (Fig. 1*A*). In *scn1Lab* mutant zebrafish, glycolysis was increased 350% from baseline to an average of 27.26 ± 1.12 mpH/min (*p* < 0.0001^e^, Fig. 1*A*). Both the immediate and delayed increase of glycolytic rates in WT and *scn1Lab* mutant zebrafish were sustained for the duration of the experiment, 48 min. After 24–32 min when *scn1Lab* mutant zebrafish had maximal response to 4-AP, the fold-change was significantly greater in *scn1Lab* mutant zebrafish than that of WT zebrafish. Behavioral seizure analysis on an independent clutch of WT larvae demonstrated that a single concentration of 4 mm 4-AP induced the severe convulsive seizure behavior (Stage III) at the 8 min time point in 23 of 48 larvae; circling Stage II behavior was noted in 25 of 48 larvae. After continuous exposure to 4-AP, seizure behavior corresponding to Stage II was observed in the majority of zebrafish larvae (47 of 48). Representative locomotion plots are shown for WT zebrafish at baseline and following exposure to 4 mm 4-AP (Fig. 1*C*). Mean velocity measurements, which act as a surrogate marker for Stage III behavior (Baraban et al., 2013) were significantly increased at the 8 min exposure point (Fig. 1*D* ^c^).

WT zebrafish showed a small, nonsignificant, increase in mitochondrial respiration immediately after 4-AP stimulation, which rapidly returned to baseline (Fig. 1*B*). However, *scn1Lab* mutant zebrafish showed a delayed and gradual increase in mitochondrial respiration with maximal increase about 30 min after stimulation. Mitochondrial respiration was increased ∼142% from baseline to 253.1 pmol/min in *scn1Lab* mutant compared with WT zebrafish.

**Figure 2.**
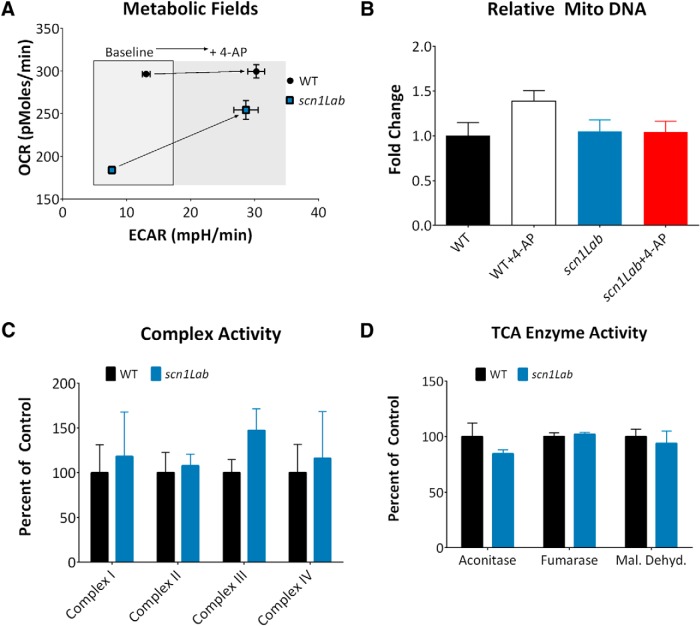
Respiratory chain complex activity in WT and *scn1Lab* mutant zebrafish. ***A***, ECAR and OCR from Figure 1 are replotted to demonstrate the metabolic field and the increases in metabolic field after treatment with 4-AP. *Scn1Lab* mutant zebrafish increase metabolism to approach the metabolic state of WT zebrafish after 4-AP, suggesting mutant zebrafish retain a similar metabolic capacity as WT zebrafish; each point represents mean±S.E.M. ***B***, Relative mitochondrial copy number was determined by total mitochondrial DNA. No significant differences were found (*n* = 3 individual animals per group; one-way ANOVA, *p* = 0.2056^gg^). ***C***, There is no difference in activity in complexes I–IV in WT and *scn1Lab* mutant zebrafish. Bars represent the mean ± SEM relative to WT activity, n = *3* groups with 25–30 fish pooled per group. Two-way ANOVA, interaction, *p* = 0.9367^hh^. ***D***, There are no differences in activity in selected TCA cycle enzymes in WT and *scn1Lab* mutant zebrafish. Bars represent the mean ± SEM relative to WT activity, *n* = 4 groups with 30 fish per group. Two-way ANOVA, interaction, *p* = 0.5801^ii^.

These data, when plotted as OCR versus ECAR, show a unique metabolic shift (Fig. 2*A*). At baseline, WT zebrafish are more glycolytic and oxidative than mutant zebrafish, but both processes are in a narrow metabolic field. Increased neuronal excitability shifts the metabolic field toward the glycolytic direction. The mutant zebrafish increase glycolysis and oxidative phosphorylation after 4-AP exposure to approach the metabolic state of WT zebrafish after 4-AP. This suggests that mutant zebrafish retain a similar metabolic capacity as WT zebrafish, or in other words, there are no defective enzymes in the downstream energetic pathway. To confirm this, we assayed three different parameters. To ensure that alterations in mitochondrial OCR were not due to differences in mitochondrial content, relative mitochondrial copy number was determined. Results demonstrate that there is no significant difference between total mitochondrial copy numbers between groups (*p* = .2056^h^; Fig. 2*B*). Next, to determine whether OCR differences were due to alterations in ETC activity, complexes I–IV were assayed in WT and *scn1Lab* mutant zebrafish. No differences were observed in the activity of any of the complexes (*p* = 0.9367^i^; Fig. 2*C*). OCR may also have been decreased in *scn1Lab* mutant zebrafish if the TCA cycle enzymes were inactive, providing fewer electron donors for the ETC. Analysis of aconitase, fumarase, and malate dehydrogenase demonstrated no difference in enzymatic activity in WT and mutant zebrafish (*p* = 0.5801^j^; Fig. 2*D*). This suggests that the primary metabolic defect may lie even further upstream, in glycolysis.

**Figure 3.**
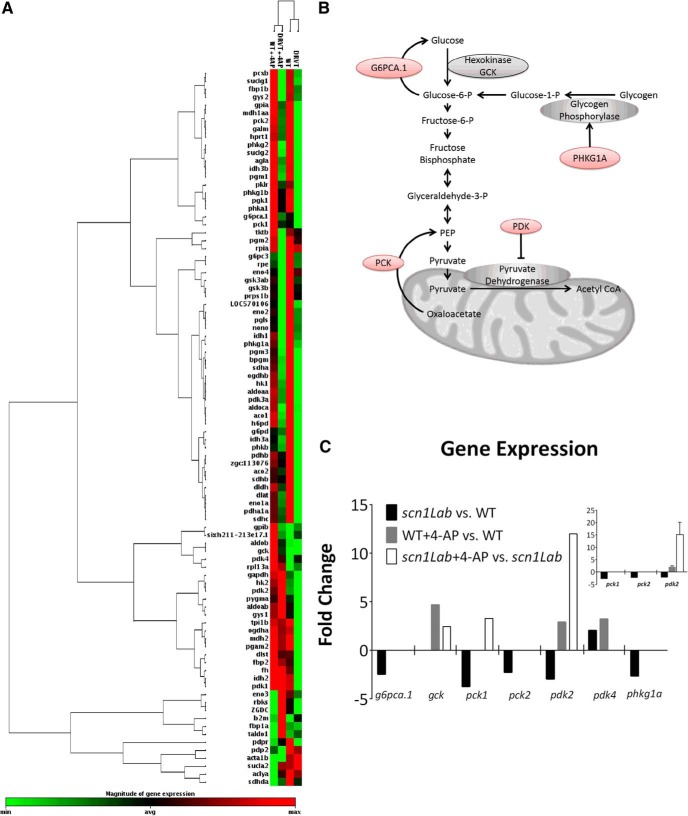
Glucose metabolism-related gene expression in WT and *scn1Lab* mutant zebrafish (DRVT) at baseline and after 4-AP stimulation. ***A***, Heatmap demonstrating relative expression of all genes analyzed from array. ***B***, Schematic depicting the pathways in which up or downregulated genes are involved. Red color indicates downregulated genes in scn1Lab mutant zebrafish vs WT at baseline. ***C***, Graph showing the seven genes with twofold or greater changes relative to respective controls. *n* = 6 pooled embryos per group analyzed once. Inset, PCR verification of genes for which specific primers were available, *pck2*, *pck4*, and *pdk2*. *N* = 3 groups of pooled embryos (6 per group) analyzed in triplicate, mean±S.E.M.

We therefore focused on the glycolytic pathway to understand the mechanistic basis of altered metabolism in *scn1Lab* mutant zebrafish. Since the activity of oxidative phosphorylation enzymes were unaltered in *scn1Lab* mutants and previous results suggested that genes related to metabolic processes are differentially expressed in *scn1Lab* mutant zebrafish (Baraban et al., 2007), we examined gene expression changes using an 84 probe glucose metabolism microarray (Fig. 3*A*,*B*). Of these, six genes were changed at least twofold (Fig. 3*C*). Five of these six were downregulated in *scn1Lab* mutant zebrafish compared with WT and included *g6pca.1*, *pck1*, *pck2*, *pdk2*, and *phkg1a*. Gene expression was also analyzed 1 h after 4-AP treatment. In 4-AP stimulated zebrafish, four genes were changed at least twofold compared with untreated controls (*gck*, *pck1*, *pdk2*, and *pdk4*). PCR array results were verified using specific Taqman primers, which were available for *pck1*, *pck2*, and *pdk2*. PCR results verified fold-changes of *pck1* (−2.83 ± 0.45), *pck2* (−2.36 ± 0.45), and *pdk2* (−2.23 ± 0.42) in *scn1Lab* versus WT zebrafish (Fig. 3*C*, inset). WT zebrafish after 4-AP stimulation were also verified to have a 2.08 ± 0.48-fold increase in *pdk2* expression; *scn1Lab* mutant zebrafish had a fold-change of 15.16 ± 4.93 in *pdk2* expression (Fig. 3*C*, inset). These results suggest that baseline decreases in ECAR and OCR in *scn1Lab* mutants likely results from mutation-induced downregulation of key glycolytic genes leading to an overall decreased flux of substrates through these pathways. Exaggerated responses in ECAR and OCR in *scn1Lab* mutants could be the result of mutation-induced upregulation of key glycolytic genes (ie, *pck1* and *pdk2*). Together, alterations in glycolytic gene expression provide a mechanistic basis for ion channel related alterations in metabolism.

**Figure 4.**
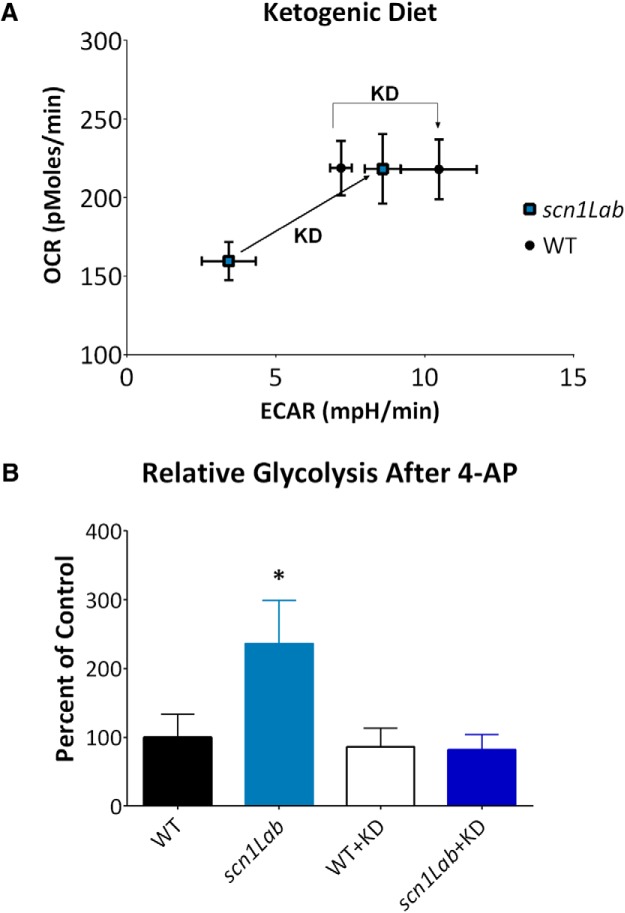
Ketogenic diet restores metabolism of *scn1Lab* mutant zebrafish to WT levels. ***A***, Metabolic profile of WT zebrafish is shifted slightly to be more glycolytic after KD treatment. Mutant zebrafish increase both glycolysis and mitochondrial respiration to WT levels. ***B***, KD treatment reduces mutant zebrafish response to 4-AP to similar to WT levels. *N* = 4 (WT), 4 (*scn1Lab*), 5 (WT+KD), 5 (*scn1Lab*+KD), *p* = .017^jj^. Values or bars indicate mean±S.E.M.

Finally, to determine whether metabolic deficits could be rescued, we exposed developing zebrafish larvae to a modified KD (Taylor et al., 2004) for 48 h. In *scn1Lab* mutant zebrafish, baseline metabolism was raised to levels similar to WT controls for both glycolysis (*p* = 0.1043^k^) and respiration (*p* = 0.9853^l^; Fig. 4*A*). 4-AP treatment produced a similar magnitude of increase in ECAR in both vehicle and KD-exposed WT controls (*p* = 0.4744^m^); however, the response to 4-AP was suppressed in the KD-exposed *scn1Lab* mutant zebrafish to the same rates as WT zebrafish (*p* = 0.1294^n^; Fig. 4*B*).

## Discussion

Here we demonstrate significant decreases in metabolism in a zebrafish model of SCN1A-related epilepsy. These mutants exhibit hyperactivity, including convulsive behavior, spontaneous electrographic seizures, shortened lifespan and a pharmacological profile similar to the human condition (Baraban et al., 2013; Dinday and Baraban, 2015). We now demonstrate, using novel methodology, that these *scn1Lab* mutant zebrafish, representing one example of a genetic epilepsy, exhibit decreased glycolytic and mitochondrial respiration rates. Although mitochondrial respiration has been measured in early embryogenesis up to 48 h postfertilization (Stackley et al., 2011) and cell-based (Giménez-Cassina et al., 2012) or rodent-based (Rowley et al., 2015) systems, this is the first time metabolism has been measured in live zebrafish larvae and could be useful in identifying metabolic dysfunction in the growing armamentarium of zebrafish mutants.

Hypometabolism in DS has not been extensively reported in the literature. However, in a single study evaluating the metabolic status of patients with DS, Craig et al. (2012) showed comorbid electron transport defects in two patients. One had impaired complex III activity, and the other complex IV, in muscle biopsies from two DS patients. The etiology of the comorbidity is not entirely obvious but the authors suggest an *scn1a* mutation is not the only cause. Our results support these suppositions. The *scn1Lab* mutant zebrafish did not show any defects in ETC complexes I–IV, suggesting respiratory chain defects may not be inherent to DS.

The mechanism by which loss of sodium-channel function results in the constellation of symptoms observed in DS is incompletely understood. Our results suggest a general decrease in expression of glycolysis related genes resulting in decreased glycolytic substrates driving mitochondrial respiration. It is possible seizures, which are spontaneously occurring in these mutants, induce oxidative stress resulting in posttranslational oxidative modification and decreased activities of complex I (Ryan et al., 2012). Metabolic dysfunction, in particular mitochondrial dysfunction, has been known to have epilepsy as a dominant or collateral feature of the phenotype (Finsterer and Zarrouk Mahjoub, 2012). In addition, mitochondrial complex I deficiency has been observed in human and experimental temporal lobe epilepsy (Kunz et al., 2000). Whether the presence of seizures alone explains the metabolic deficits is unclear and needs further exploration.

Interestingly and consistent with a metabolic defect, KDs have shown efficacy in controlling seizures in some DS patients (Caraballo, 2011) and the *scn1Lab* mutant zebrafish used here (Baraban et al., 2013). We now show that a KD also improves the metabolic state of *scn1Lab* mutant zebrafish to WT levels. One potential mechanism of action is that the KD alters expression of glucose-related metabolism genes in *scn1Lab* mutant zebrafish to WT levels. However, this seems unlikely because alteration of the genes reported here, and others, by KD has not been reported in the literature. Murata et al. (2013) fed WT and *Fgf21* (fibroblast growth factor) knockout mice KD and showed no difference in *pck* expression, or glucose-6-phosphatase and PPARɣ coactivator-1α, compared with normal diet. Jornayvaz et al. (2010) showed similar results in KD fed mice for *pck*, glucose-6-phosphatase, and pyruvate carboxylase compared to control diet. However, a more likely mechanism is that a KD alters the fuels available for glycolysis and oxidative phosphorylation to use. Impairment of mitochondrial bioenergetics capacity can critically affect apoptosis, neuronal excitability, and seizure susceptibility (Gano et al., 2014). Ketone bodies can decrease the spontaneous firing of GABAergic neurons, dependent on K_ATP_ channels (Ma et al., 2007) and given the deficits in interneuron function thought to be associated with *scn1a* mutation this could be a direct site of action.

4-AP is a potassium channel blocker that is commonly used to elicit seizures in animal models, including larval zebrafish (Baraban et al., 2007). Here, we demonstrate that WT zebrafish respond to 4-AP by immediately increasing glycolysis but not mitochondrial respiration corresponding with severe behavioral seizures. The metabolic alteration is reminiscent of ictal hypermetabolism (Stafstrom et al., 2008) and it is interesting to note that *scn1Lab* mutants have delayed increases in glycolysis and mitochondrial respiration after a 4-AP challenge. We also report a significant downregulation of phosphoenolpyruvate carboxykinases (*pck1* and *pck2*) and pyruvate dehydrogenase kinase (*pdk2*) in *scn1Lab* mutant, genes important in gluconeogenesis, glycogenolysis, and regulation of pyruvate dehydrogenase. Specifically, *pck* is responsible for converting oxaloacetate in to phosphoenolpyruvate, a glycolysis intermediary and its loss would result in less intermediates and less glycolytic flux. *Pdk2* is a gene responsible for inhibition of pyruvate dehydrogenase, which converts pyruvate to acetyl CoA. Decreased *pdk2* would result in increased pyruvate dehydrogenase activity and less pyruvate and lactate. This could explain the lower ECAR observed here (Sun et al., 2011). It may appear that increased pyruvate dehydrogenase would result in increased respiration in *scn1Lab* mutant zebrafish. However, because there are fewer intermediates, and less flux through glycolysis, it is reasonable that the overall respiration rate is reduced in *scn1Lab* mutant zebrafish. In WT zebrafish, the increase in *pdk2* would increase inhibition of pyruvate dehydrogenase and subsequently lead to increases in glycolytic metabolites pyruvate and lactate, suggesting a mechanism for increased glycolysis.

**Figure 5.**
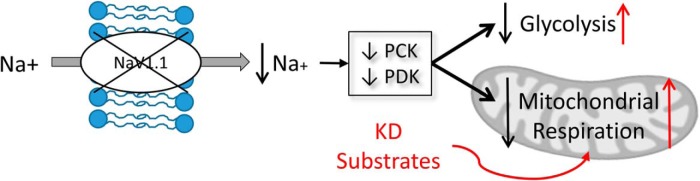
Summary diagram depicting proposed mechanism. Mechanism demonstrating changes in glycolysis and mitochondrial respiration in *scn1* mutant zebrafish at baseline (in black) and proposed action of KD (in red).

Overall, our results suggest a metabolic impairment concomitant with a sodium channel mutation in DS, likely due to glycolysis gene-expression changes, and reversible by the KD (Fig. 5). We demonstrate this with a novel technique to assess ECAR and OCR in live zebrafish and propose that the metabolic impairment in *scn1Lab* mutant zebrafish may be due to changes in gene expression related to glucose metabolism (Fig. 3*B*). This approach was applied here to one genetic model of epilepsy, but opens new avenues for studying metabolism in any zebrafish model or as a means to screen compound libraries for new drugs that would improve metabolic function.

**Figure 6.**
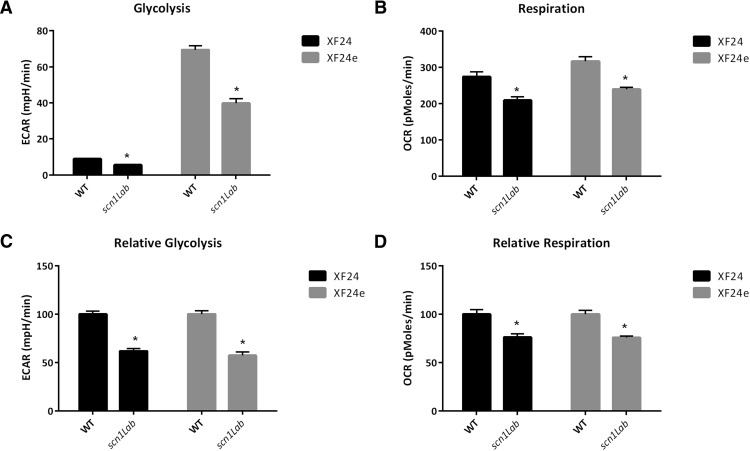
Metabolic differences in WT and *scn1lab* mutant zebrafish are reproducible when grown in a separate facility, as well as with different instrumentation. All zebrafish in this figure were bred and grown at UCD. Using the same instrumentation as Figures 1 and 4 (XF24) the baseline differences are recapitulated in zebrafish grown at UCSF. The newer model of extracellular flux analysis (XF24e) is more sensitive and thus has higher baseline values for both glycolysis and mitochondrial respiration. ***A***, ***B***, Absolute baseline differences in glycolysis and respiration are recapitulated in both the XF24 and newer XF24e. ***A***, XF24, *p* < .000^kk^; XF24e, *p* < .00^ll^; ***B***, XF24, *p* = .00^mm^; XF24e, *p* < .00^nn^. ***C***, ***D***, Although the XF24e is more sensitive, the relative differences in glycolysis and respiration are similar in both the XF24 and XF24e. ***C***, XF24, *p* < .00^oo^; XF24e, *p* < .00^pp^. ***D***, XF24, *p* = .0002^qq^; XF24e, *p* < .0001^rr^. Bars represent mean±S.E.M.

## Synthesis

The decision was a result of the Reviewing Editor Elizabeth Powell and the peer reviewers coming together and discussing their recommendations until a consensus was reached. A fact-based synthesis statement explaining their decision and outlining what is needed to prepare a revision is listed below. The following reviewers agreed to reveal their identity: Annapurna Poduri, Timothy Simeone

Summary of NWR-0008-16

This submission describes metabolic consequences in the absence of the zebrafish ortholog of the Dravet syndrome gene, Scn1Lab. Both reviewers are enthusiastic about the study and agree that the work advances the field. However, there are several items that need to be addressed before the submission is appropriate for publication. In particular, the clinical relevance should be modified and the PCR array methods and results need to be better explained and included.

Reviewer #1

Advances the field: New techniques are presented that expand the repertoire of analyses that can be applied to zebrafish models of epilepsy and potentially other disorders.

Review - The authors provide an interesting new experimental paradigm to assess glycolysis and mitochondrial enzyme activity, TCA activity, and related gene expression in a zebrafish larval system. These techniques are worthy of reporting. The model used, the Scn1Lab, is an established and published zebrafish model of epilepsy.

What is not clear to this reviewer is how much of the effect seen in the Scn1Lab larvae is specifically related to SCN1A protein dysfunction vs a general inherent tendency to epilepsy that would be seen in any genetic model of epilepsy in the zebrafish in which spontaneous epileptiform activity would be expected to occur, at least episodically. Ideally, as more genetic models of epilepsy become available, it would make sense to use the array of techniques demonstrated in this manuscript to compare different models.

In addition to concern about the specificity of the effects seen to Scn1a in the zebrafish, I have concerns about the lengthy introduction and discussion of Dravet syndrome in humans. The evidence for hypometabolism in Dravet syndrome is not strong, and in fact, we do not know the means by which the ketogenic diet appears to be successful in some patients with Dravet syndrome. Some of the references to ictal hypometabolism cited refer to findings in patients with focal epilepsy (NOT Dravet syndrome). As the authors do point out, the mitochondrial abnormalities noted, e.g., complex III and IV abnormalities, are not specific to patients with sodium channel mutations.

Thus, I would recommend the following:

(1) reducing the emphasis on the clinical relevance of these findings in both the introduction and discussion, including the removal of references that are not directly relevant,

(2) presenting the data as ONE example of features that can be assessed in genetic models of zebrafish, minimizing a specific role of SCN1A unless more data can be provided to support it,

and

(3) providing examples of such methods used in other systems (e.g., cell-based or rodent) if they are available with a rationale for why they are applicable and useful in the zebrafish system.

Reviewer #2

Advances the field: This manuscript provides evidence for potential metabolic dysfunction in Dravet syndrome that should be tested in the future for confirmation. Furthermore, this study sets the stage for identification of metabolic dysfunction in other genetic epilepsies and performing high-throughput screening for metabolic therapies.

Review - This manuscript presents a unique and exciting study on metabolic dysfunction in a zebrafish model of Dravet syndrome. The authors find that zebrafish carrying a mutation in scn1a have reduced glycolysis and mitochondrial respiration rates. These functional differences were not correlated with changes in function of individual complexes in the ETC or enzymes in the TCA cycle. They were associated with down-regulation of genes involved in glucose metabolism. Acute 4-AP application resulted in increases in glycolysis, respiration rates and up-regulation of genes. Speculating that bypassing glucose metabolism may be beneficial, the authors' fed the scn1a mutant zebrafish a ketogenic diet and glycolysis and respiration were rescued to wild-type levels. This manuscript provides evidence for potential metabolic dysfunction in Dravet syndrome that should be tested in the future for confirmation. Furthermore, this study sets the stage for identification of metabolic dysfunction in other genetic epilepsies and performing high-throughput screening for metabolic therapies.

This study is well designed and strong, however, I have listed a few concerns below that need to be addressed. Most importantly the authors' need to address the questions concerning the PCR array experiment and develop the discussion section a bit more.

1. Abstract: the abstract ends abruptly without any interpretation or conclusion.

Results: Why switch from PTZ to 4-AP for the chronic and acute experiments? Because the authors' demonstrate acute effects of 4-AP on OCR and ECAR and genes, the conclusions about the non-effect of chronic seizures would be strengthened by chronic 4-AP experiments.

2. Results: Please provide SEM when stating averages in the text.

3. Results: The paragraph beginning line 160 seems redundant with the previous paragraph.

4. Results: Figs 1E and F are unnecessary.

5. Results, line 165, and Fig 2a: For the OCR v. ECAR graph which time point corresponds to the values used to construct the graph. No matter which time point, I would expect the WT+4AP ECAR to be over 30.

6. Results: How long were the fish exposed to 4-AP for the mitochondria copy number and PCR array experiments?

7. Methods, Results, Fig 3: The way the PCR array methods and data is presented it appears that 6 zebrafish were pooled for each sample giving an n=1 per group which explains the lack of error bars in Fig. 3c. What was the variation in the replicates? If it is an n=1 per group can the authors truly draw any conclusions? Please show the PCR results verifying the array results.

8. Fig 3b: Please indicate which genes are up or down regulated.

9. Methods: Please describe the KD treatment.

10. Results, Fig 4: Which experimental time point corresponds to the OCR and ECAR values? Please present the data over the experimental time course as in Fig 1A and B. Why are the baseline values so different from Fig. 2a?

11. Discussion: Please reference and discuss figure 5.

12. Discussion, paragraph beginning line 242: The authors describe "one potential mechanism" of the KD as providing glycolytic substrates. What are other potential mechanisms? Specifically, does the KD change the expression of the genes described in this study? Or any other genes involved in glycolysis? How does this relate to the authors' findings?

13. Discussion: The authors' need to discuss the PCR array findings a bit more. As one of the major findings of the paper a more in depth discussion/speculation is warranted. Specifically, an explicit description of how/why down regulation of enzymes in glucose metabolism would affect glycolysis and mitochondrial respiration rates. Likewise, how does upregulation by 4-AP lead to increases of glycolysis and respiration. What does this mean for glucose utilization in the different genotypes/treatments? Furthermore, how do these differences relate to acute seizures (or is 4-AP more like status epilepticus) and chronic, spontaneous recurrent seizures?

14. Discussion, final paragraph: Please take out the reference to the Seahorse analyzer. This is a tool or a means to an end. It is not the focus of the manuscript.

15. Fig 6: This figure seems to be left over from a previous draft of this manuscript and is not mentioned in the results. Please explain.

## References

[B1] Artuso L, Romano A, Verri T, Domenichini A, Argenton F, Santorelli FM, Petruzzella V (2012) Mitochondrial DNA metabolism in early development of zebrafish (Danio rerio). Biochim Biophys Acta 1817:1002-1011. 10.1016/j.bbabio.2012.03.019 22465854

[B2] Baraban SC, Taylor MR, Castro PA, Baier H (2005) Pentylenetetrazole induced changes in zebrafish behavior, neural activity and c-Fos expression. Neuroscience 131:759-768. 10.1016/j.neuroscience.2004.11.031 15730879

[B3] Baraban SC, Dinday MT, Hortopan GA (2013) Drug screening in Scn1a zebrafish mutant identifies clemizole as a potential Dravet syndrome treatment. Nat Commun 4:2410. 10.1038/ncomms3410 24002024 PMC3891590

[B4] Baraban SC, Dinday MT, Castro PA, Chege S, Guyenet S, Taylor MR (2007) A large-scale mutagenesis screen to identify seizure-resistant zebrafish. Epilepsia 48:1151-1157. 10.1111/j.1528-1167.2007.01075.x 17521353 PMC2211740

[B5] Caraballo RH (2011) Nonpharmacologic treatments of Dravet syndrome: focus on the ketogenic diet. Epilepsia 52:79-82. 10.1111/j.1528-1167.2011.03009.x 21463287

[B6] Chugani HT, Rintahaka PJ, Shewmon DA (1994) Ictal patterns of cerebral glucose utilization in children with epilepsy. Epilepsia 35:813-822. 8082628 10.1111/j.1528-1157.1994.tb02517.x

[B7] Craig AK, de Menezes MS, Saneto RP (2012) Dravet syndrome: patients with co-morbid SCN1A gene mutations and mitochondrial electron transport chain defects. Seizure 21:17-20. 10.1016/j.seizure.2011.08.010 21906962

[B8] Dinday MT, Baraban SC (2015) Large-scale phenotype-based antiepileptic drug screening in a zebrafish model of Dravet syndrome(1,2,3). eNeuro 2:ENEURO.0068-15.2015. 10.1523/ENEURO.0068-15.2015 26465006 PMC4596025

[B9] Dravet C (2011) The core Dravet syndrome phenotype. Epilepsia 52:3-9. 10.1111/j.1528-1167.2011.02994.x 21463272

[B10] Finsterer J, Zarrouk Mahjoub S (2012) Epilepsy in mitochondrial disorders. Seizure 21:316-321. 10.1016/j.seizure.2012.03.003 22459315

[B11] Flynn JM, Choi SW, Day NU, Gerencser AA, Hubbard A, Melov S (2011) Impaired spare respiratory capacity in cortical synaptosomes from Sod2 null mice. Free Radic Biol Med 50:866-873. 10.1016/j.freeradbiomed.2010.12.030 21215798 PMC3061438

[B12] Gano LB, Patel M, Rho JM (2014) Ketogenic diets, mitochondria, and neurological diseases. J Lipid Res 55:2211-2228. 10.1194/jlr.R048975 24847102 PMC4617125

[B13] Giménez-Cassina A, Martinez-François JR, Fisher JK, Szlyk B, Polak K, Wiwczar J, Tanner GR, Lutas A, Yellen G, Danial NN (2012) BAD-dependent regulation of fuel metabolism and K(ATP) channel activity confers resistance to epileptic seizures. Neuron 74:719-730. 10.1016/j.neuron.2012.03.032 22632729 PMC3361694

[B14] Grisar T (1984) Glial and neuronal Na+-K+ pump in epilepsy. Ann Neurol 16:S128-S134. 6095737 10.1002/ana.410160719

[B15] Hunter SE, Jung D, Di Giulio RT, Meyer JN (2010) The QPCR assay for analysis of mitochondrial DNA damage, repair, and relative copy number. Methods 51:444-451. 10.1016/j.ymeth.2010.01.033 20123023 PMC2912960

[B16] Jornayvaz FR, Jurczak MJ, Lee HY, Birkenfeld AL, Frederick DW, Zhang D, Zhang XM, Samuel VT, Shulman GI (2010) A high-fat, ketogenic diet causes hepatic insulin resistance in mice, despite increasing energy expenditure and preventing weight gain. Am J Physiol Endocrinol Metab 299:E808-E815. 10.1152/ajpendo.00361.2010 20807839 PMC2980360

[B17] Kunz WS, Kudin AP, Vielhaber S, Blümcke I, Zuschratter W, Schramm J, Beck H, Elger CE (2000) Mitochondrial complex I deficiency in the epileptic focus of patients with temporal lobe epilepsy. Ann Neurol 48:766-773. 11079540

[B18] Lee EM, Park GY, Im KC, Kim ST, Woo CW, Chung JH, Kim KS, Kim JS, Shon YM, Kim YI, Kang JK (2012) Changes in glucose metabolism and metabolites during the epileptogenic process in the lithium-pilocarpine model of epilepsy. Epilepsia 53:860-869. 10.1111/j.1528-1167.2012.03432.x 22429025

[B19] Liang LP, Waldbaum S, Rowley S, Huang TT, Day BJ, Patel M (2012) Mitochondrial oxidative stress and epilepsy in SOD2 deficient mice: attenuation by a lipophilic metalloporphyrin. Neurobiol Dis 45:1068-1076. 10.1016/j.nbd.2011.12.025 22200564 PMC3418969

[B20] Ma W, Berg J, Yellen G (2007) Ketogenic diet metabolites reduce firing in central neurons by opening K(ATP) channels. J Neurosci 27:3618-3625. 10.1523/JNEUROSCI.0132-07.2007 17409226 PMC6672398

[B21] Ma YY, Zhang XL, Wu TF, Liu YP, Wang Q, Zhang Y, Song JQ, Wang YJ, Yang YL (2011) Analysis of the mitochondrial complex I-V enzyme activities of peripheral leukocytes in oxidative phosphorylation disorders. J Child Neurol 26:974-979. 10.1177/0883073811399905 21540367

[B22] Mecocci P, MacGarvey U, Kaufman AE, Koontz D, Shoffner JM, Wallace DC, Beal MF (1993) Oxidative damage to mitochondrial DNA shows marked age-dependent increases in human brain. Ann Neurol 34:609-616. 10.1002/ana.410340416 8215249

[B23] Murata Y, Nishio K, Mochiyama T, Konishi M, Shimada M, Ohta H, Itoh N (2013) Fgf21 impairs adipocyte insulin sensitivity in mice fed a low-carbohydrate, high-fat ketogenic diet. PloS One 8:e69330. 10.1371/journal.pone.0069330 23874946 PMC3706421

[B24] Patel M, Day BJ, Crapo JD, Fridovich I, McNamara JO (1996) Requirement for superoxide in excitotoxic cell death. Neuron 16:345-355. 8789949 10.1016/s0896-6273(00)80052-5

[B25] Rowley S, Liang LP, Fulton R, Shimizu T, Day B, Patel M (2015) Mitochondrial respiration deficits driven by reactive oxygen species in experimental temporal lobe epilepsy. Neurobiol Dis 75:151-158. 10.1016/j.nbd.2014.12.025 25600213 PMC4465449

[B26] Ryan K, Backos DS, Reigan P, Patel M (2012) Post-translational oxidative modification and inactivation of mitochondrial complex I in epileptogenesis. J Neurosci 32:11250-11258. 10.1523/JNEUROSCI.0907-12.2012 22895709 PMC3518304

[B27] Stackley KD, Beeson CC, Rahn JJ, Chan SS (2011) Bioenergetic profiling of zebrafish embryonic development. PloS One 6:e25652. 10.1371/journal.pone.0025652 21980518 PMC3183059

[B28] Stafstrom CE, Roopra A, Sutula TP (2008) Seizure suppression via glycolysis inhibition with 2-deoxy-D-glucose (2DG). Epilepsia 49:97-100. 10.1111/j.1528-1167.2008.01848.x 19049601

[B29] Sun W, Chang SS, Fu Y, Liu Y, Califano JA (2011) Chronic CSE treatment induces the growth of normal oral keratinocytes via PDK2 upregulation, increased glycolysis and HIF1α stabilization. PloS One 6:e16207. 10.1371/journal.pone.0016207 21283817 PMC3023770

[B30] Taylor MR, Hurley JB, Van Epps HA, Brockerhoff SE (2004) A zebrafish model for pyruvate dehydrogenase deficiency: rescue of neurological dysfunction and embryonic lethality using a ketogenic diet. Proc Natl Acad Sci U S A 101:4584-4589. 10.1073/pnas.0307074101 15070761 PMC384790

[B31] Tenney JR, Rozhkov L, Horn P, Miles L, Miles MV (2014) Cerebral glucose hypometabolism is associated with mitochondrial dysfunction in patients with intractable epilepsy and cortical dysplasia. Epilepsia 55:1415-1422. 10.1111/epi.12731 25053176

[B32] Tibbs GR, Barrie AP, Van Mieghem FJ, McMahon HT, Nicholls DG (1989) Repetitive action potentials in isolated nerve terminals in the presence of 4-aminopyridine: effects on cytosolic free Ca2+ and glutamate release. J Neurochem 53:1693-1699. 2553862 10.1111/j.1471-4159.1989.tb09232.x

[B34] Wallace DC, Zheng XX, Lott MT, Shoffner JM, Hodge JA, Kelley RI, Epstein CM, Hopkins LC (1988) Familial mitochondrial encephalomyopathy (MERRF): genetic, pathophysiological, and biochemical characterization of a mitochondrial DNA disease. Cell 55:601-610. 3180221 10.1016/0092-8674(88)90218-8

[B35] Wu M (2009) Real-time measurement of mitochondrial respiration and glycolysis rates of cancer cells in a microplate AACR Education Book 2009 pp. 295-300.

